# C-reactive protein binds to short phosphoglycan repeats of *Leishmania* secreted proteophosphoglycans and activates complement

**DOI:** 10.3389/fimmu.2023.1256205

**Published:** 2023-08-31

**Authors:** Eu Shen Seow, Eve C. Doran, Jan-Hendrik Schroeder, Matthew E. Rogers, John G. Raynes

**Affiliations:** Department of Infection Biology, Faculty of Infectious and Tropical Diseases, London School of Hygiene and Tropical Medicine, London, United Kingdom

**Keywords:** C-reactive protein, proteophosphoglycan, Leishmania, complement, promastigote secretory gel, secreted acid phosphatase

## Abstract

Human C-reactive protein (CRP) binds to lipophosphoglycan (LPG), a virulence factor of *Leishmania* spp., through the repeating phosphodisaccharide region. We report here that both major components of promastigote secretory gel (PSG), the filamentous proteophosphoglycan (fPPG) and the secreted acid phosphatase (ScAP), are also ligands. CRP binding was mainly associated with the flagellar pocket when LPG deficient *Leishmania mexicana* parasites were examined by fluorescent microscopy, consistent with binding to secreted material. ScAP is a major ligand in purified fPPG from parasite culture as demonstrated by much reduced binding to a ScAP deficient mutant fPPG in plate binding assays and ligand blotting. Nevertheless, in sandfly derived PSG fPPG is a major component and the major CRP binding component. Previously we showed high avidity of CRP for LPG ligand required multiple disaccharide repeats. ScAP and fPPG only have short repeats but they retain high avidity for CRP revealed by surface plasmon resonance because they are found in multiple copies on the phosphoglycan. The fPPG from many species such as *L. donovani* and *L. mexicana* bound CRP strongly but *L. tropica* and *L. amazonensis* had low amounts of binding. The extent of side chain substitution of [-PO_4_-6Galβ1-4Manα1-] disaccharides correlates inversely with binding of CRP. The ligand for the CRP on different species all had similar binding avidity as the half maximal binding concentration was similar. Since the PSG is injected with the parasites into host blood pools and phosphoglycans (PG) are known to deplete complement, we showed that CRP makes a significant contribution to the activation of complement by PSG using serum from naive donors.

## 1 Introduction

Leishmaniasis is a zoonotic disease caused by the *Leishmania* genus of protozoan parasites and a major neglected tropical disease, with 500,000 to 900,000 estimated new cases and 18,700 fatalities per year (1). It affects diverse mammalian species and is transmitted by female Phlebotominae sand flies during bloodmeal feeding attempts.

During the insect stage and during the infection of mammalian hosts the parasites produce a variety of phosphoglycan structures that aid in their survival and virulence (2). These include the relatively well studied membrane-associated lipophosphoglycan (LPG) ligand. However, parasites also produce proteophosphoglycans (PPG) including filamentous proteophosphoglycan (fPPG) and secreted acid phosphatase (ScAP) in the infected sand fly. The secreted PPGs are introduced into the feeding site as part of the promastigote secretory gel (PSG) alongside mammalian-infectious metacyclic promastigotes and sand fly saliva. Studies using knock out of LPG1 or LPG2 (3) have shown a complicated virulence phenotype differing in different parasites, for instance, LPG deficiency in *Leishmania major* (4) and *Leishmania infantum* (5) suggests virulence in mammalian infection studies but *Leishmania mexicana* did not (3, 6).

Proteophosphoglycans share similar glycan moieties to LPG, as shown by the cross-reactivity of many antibodies between LPG and the PSG components fPPG and ScAP (7). ScAP and fPPG have high carbohydrate contents, which form more than 70% of their molecular weight. While their exact function is currently unknown, PSG injected with or without saliva promotes cutaneous leishmaniasis infection in murine studies, suggesting that PSG may be a virulence factor (8, 9). The main component of PSG and PPG is fPPG, a mucin-like molecule. The types and structures of the phosphoglycans show some similarities but also significant differences between species in terms of when expressed, glycan content and whether secreted or membrane bound. ScAP, while not the primary constituent of PPG, is associated with fPPG and the subunits are hard to dissociate from the polymer structure (10). The potential role of PSG in establishing human infection remains an underreported field of study, particularly in light of the likely importance of the early immune response to the determination of Leishmaniasis outcome.

Among possible innate recognition molecules, CRP has been shown to bind to *Leishmania* surface LPG (11). Classically CRP is known to bind in a calcium dependent way to phosphorylcholine (PCh) which is a common component in a variety of fungal, bacterial and parasitic products. In this manner, CRP acts as an important innate immune activator of the classical complement pathway via CRP (12, 13). In the case of *Leishmania*, CRP binds with high affinity in a calcium dependent manner to the phosphorylated galactose mannose disaccharide repeat found in *Leishmania* LPG (11). High affinity interactions required between 3 and 10 repeats of [-PO4-6Galβ1-4Manα1-] when provided as chemically synthesised soluble form (14). This interaction with LPG has biological relevance for a number of reasons. It can lead to increased uptake into macrophages under physiological conditions, although this may not lead to increased killing but rather aid the parasite in establishing infection (15). In addition, the binding of the CRP to the surface of the parasite has also been shown to help the parasite initiate transformation in *L. mexicana* (16) and *L. donovani* (17). CRP interaction with LPG thus has importance in infection of the mammalian host from the sand fly.

This report now investigates the potential interaction of CRP with the PSG phosphoglycans. Whilst the longer LPG from metacyclic parasites shows greatly increased binding to CRP compared to ‘non-infectious log phase promastigotes’ both these have much greater length than that reported for the phosphoglycans of ScAP and proteophosphoglycans that have typically an average of 2-3 repeats (10). Individual soluble synthetic structures were shown to have only weak ability to interact with CRP (14). However, we demonstrate here that the interaction of CRP with proteophosphoglycan is high avidity when these short repeats of [-PO4-6Galβ1-4Manα1-] are presented in multiple sites on the extended structure.

One way in which the interaction of CRP might alter infection rates and/or survival of the parasite is through the complement pathway. Importantly CRP binding of a ligand does not inevitably lead to complement activation as seen recently for a phosphorylcholine substituted glycoprotein ES-62 (18). The LPG is a known activator of complement particularly to extended LPG in metacyclic forms causing deposition of C3b but without damaging the parasite (19). This is in contrast to logarithmic phase parasites which are all rapidly killed by complement. It is already known that a potentially important role of secreted PPGs in PSG such as fPPG and ScAP is the activation and depletion of complement. PPG injected into a mouse could dramatically deplete complement by 90% within 30 minutes (20). The potential mechanisms of this could be classical or lectin pathway mediated as the activation was calcium dependent but a role for CRP was not assessed. Here we show that using naive donor sera CRP makes a significant contribution to the total complement activation by the proteophosphoglycan thereby implicating a role in complement depletion.

## 2 Methods

### 2.1 Purification of proteophosphoglycan

Parasites of *Leishmania mexicana* (MNYC/BZ/62/M379); *Leishmania major* (LV39; (MRHO/SU/59/P); *Leishmania donovani* (MHOM/ET/67/HU3); *Leishmania panamenensis* (MHOM/PA/67/BOYNTON); *Leishmania infantum* (MHOM/BR/76/M4192); *Leishmania amazonensis* (LV79: MPRO/BR/72/M1841); *Leishmania aethiopica* (MHOM/ET/84/KH); *Leishmania tropica* (MHOM/AF/2015/HTD7) were cultured as described (21). Lipophosphoglycan (LPG) and phosphoglycan deficient *L. mexicana* were a kind gift from Dr Thomas Ilg. The LPG-deficient mutant (*lpg1−/–*) lacks the LPG1 gene, which encodes a galactofuranosyltransferase required for synthesis of the LPG glycan core, rendering them deficient in LPG alone whilst *L mexicana lpg2-/-* lacks all repeating phosphodisaccharide. Selection antibiotics hygromycin (20 μg/ml) and phleomycin (2.5 μg/ml) were added to the culture medium (3, 6) for deficient *L mexicana* mutants *lpg1-/-* and *lpg2 -/-*. *L. mexicana* (Δ*lmScAP*1/2) and an add back of ScAP2 were a kind gift of Dr Wiese (22) which employed a similar selection antibiotic pressure but including G418 (10µg/ml) in the add back.

Culture supernatants were clarified by centrifugation at low speed (800g) and passed through an anion exchange column (DE52) equilibrated in 20 mM Tris-HCl, 100 mM NaCl, pH 7.5 at a rate of 2 ml/min and eluted in 20 mM Tris-HCl, pH 7.5 containing 500 mM NaCl (23). Following anion exchange, material was pelleted by ultracentrifugation at 100,000 g, 4°C for 4 hours in a Ti90 rotor and the pellet washed and resuspended in PBS.

A further step to remove more hydrophobic fractions such as LPG used hydrophobic interaction chromatography. Pelleted PPG was adjusted to 1 M ammonium sulphate via dilution with concentrated ammonium sulphate solution (10 M NH_2_SO_4_, 1:9 v/v ratio relative to material and added to a 5 ml column of octyl-Sepharose equilibrated in 20 mM Tris-HCl, 1 M NH_2_SO_4_, 5 mM EDTA, pH 7.5. This was followed by low ionic strength Tris-buffered saline (10 ml, 20 mM Tris-HCl, 100 mM NaCl, pH 7.5), Tris-buffered saline (20 ml, 20 mM Tris-HCl, pH 7.5) and then propan-1-ol (20%, 70% v/v) which eluted more hydrophobic glycans such as LPG. Quantification was based on A280 (Nanodrop) which correlated with the carbohydrate assay using the phenol-sulphuric acid method (24). PPG material was dialysed and stored at -80°C in 20 µl aliquots. The purified PPG was confirmed to be high molecular weight (above 100kD) and heterogeneous by SDS PAGE and detection by Stains-all and confirmed as phosphoglycan using Western blotting with CA7AE (22).

The biotinylation of PPG was performed using a method using NHS-LC-biotin in pH 9.6 carbonate buffer described previously (23). Labelled material was repurified by anion exchange on DE-52 as used for purification with extensive washing to remove unreacted biotin. Plate assays showed biotin-PPG and PPG had similar CRP binding dose response (data not shown).

### 2.2 CRP and biotinylated CRP

Human CRP and recombinant CRP were purified as described previously (18). Rat CRP was purified using phosphorylcholine (PCh) Sepharose by the same protocol. CRP was biotinylated at a neutral pH as described for the reagent NHS-LC-biotin (Thermofisher) to prevent CRP denaturation. The CRP biotin was repurified on PCh-Sepharose to remove non-binding protein with extensive washing to remove unreacted biotin.

### 2.3 PPG-pentraxin plate binding assays

Purified PPG (0-3 µg/ml) in PBS pH 7.4 was coated onto 96 well microtiter plates (Immulon™ 2 HB 96-Well Microtiter EIA Plate, ImmunoChemistry Technologies, LLC). Biotinylated and affinity purified CRP (0-3 µg/ml) in HBSC-BSA was added and the plates were incubated at room temperature for 1 hour. For inhibition assays, PCh chloride (Sigma-Aldrich) or EDTA (10 mM) or MgCl_2_ (0.5 mM) and 10 mM EGTA or CRP depleted serum (5% v/v) was added at the CRP incubation stage. After washing with HBSC, the bound CRP was detected with streptavidin HRP (Biosource diluted 1 in 15,000) and 3,3′,5,5′-Tetramethylbenzidine (TMB, 0.1 mg/ml) in phosphate-citrate buffer (0.05 M, pH 5.0) with hydrogen peroxide (0.006% v/v) added following an HRP-conjugated antibody incubation stage. The subsequent colour change reaction was stopped with H_2_SO_4_ (2 M, 15 µl). The plate was read at wavelength 450 nm with subtraction of the reference wavelength at 405 nm (Titertek Multiskan MCC/340). Alternatively native pure CRP was used and detected with primary antibody rabbit anti-CRP (Dako: 1:800) followed by HRP-conjugated Goat anti-rabbit IgG (Bio-Rad, 1:3000) in HBSC-BSA. Mouse CRP binding was examined using the same buffer system and plates but using mouse CRP and affinity purified goat anti-mouse CRP (both R and D).

### 2.4 Western/ligand blot and protein gel staining

SDS-PAGE was performed using an extended 4% stacking gel layer in combination with a 6% or 10% resolving gel layer with reducing conditions. The molecular weight standards used were prestained, broad range (10-250 kDa, New England Biolabs).

Gels were then transferred by semi-dry blotting to PVDF membranes. Membranes were blocked with PBST containing 2% (w/v) BSA at 4°C o/n. Membranes were incubated with CRP biotin (1 µg/ml) or CRP (as shown in Figures) in TBST containing 0.5 mM CaCl_2_ and 1% (w/v) BSA for 1 hour at room temperature and after 3 washes in the same buffer, bands where CRP bound were visualised with streptavidin alkaline phosphatase (AP) (1 in 500; SA5100 Vector labs) or anti-CRP-AP and colorimetric detection with substrate BCIP/NBT as described (18). Detection of phosphoglycan repeated disaccharide was performed using 1:1000 CA7AE (GeneTex) then followed by 1:30,000 anti-mouse IgM-AP (Sigma).

### 2.5 Surface plasmon resonance

Polycarboxylate chips with high charge density (Xantec, C30M) were washed and activated with EDC and NHS for 7 minutes. 0.5 mM aminodesthiobiotin in 10 mM sodium maleate buffer pH 6.8 was added at a flow rate of 2 µl/min for 40 minutes to give 150 RU attached. Reactive groups on both test and control surfaces were blocked with 1 M ethanolamine pH 8.0. Neutravidin (10 μg/ml) in calcium-containing HEPES buffer (HBSPC) was then immobilised on the desthiobiotin surface of both test and control surface to give a further 150 RU. Biotin labelled purified fPPG was then added at 20 µg/ml till 220 RU was attached to the test well only. CRP was flowed over at 30 µl/min for 3 minutes at various concentrations in HBSPC buffer containing 0.5 mM CaCl_2_ and dissociation assessed for 5 minutes before CRP was dissociated with HBS containing 10 mM EDTA. In other experiments, inhibitors were added with the CRP or other proteins. Between each addition, the chip was washed with HBS containing 10 mM EDTA.

Analysis was performed using BiaEval 4.1.1. Control flow cells were subtracted and traces overlain. Kinetic data was obtained using simultaneous ka/kd 1:1 Langmuir analysis which achieved a good fit at low ligand immobilization.

### 2.6 Serum CRP depletion

Carboxylated Magnetic beads (Mobitec-beads, 100μl) were washed into PBS buffer. N-(3-Dimethylaminopropyl)-N′-ethylcarbodiimide (10 µl, 10 mg/ml) was added to the beads and left to incubate at room temperature on a rocker for 5 minutes. Chicken anti-CRP IgG antibody (1 mg/ml, 25 µl, Norwegian Antibodies) was added and left to incubate and cross-link overnight at room temperature. Blocking of remaining active cross-linking sites was achieved by incubation in 0.1 M glycine buffer (pH 8.3) for 1 hour at room temperature. Beads were washed into phosphate-buffered saline pH 7.4 with BSA (1% w/v) then washed into HEPES-buffered saline (20 mM HEPES, 150 mM NaCl, pH 7.4) with 0.5 mM CaCl_2_ (HBSC) and stored at 4°C. Whole serum from normal, healthy donors collected under ethical approval (100 µl) was mixed with the anti-CRP beads and allowed to incubate on a rocker at 2°C for an hour immediately prior to experiments. Typical reduction in CRP concentrations were 50-95% determined using a sandwich ELISA for CRP (25).

### 2.7 Complement assays

#### 2.7.1 C1q capture assay

An immune complex capture assay (18) was used to measure complex formation between CRP and different CRP ligands, including PPG or control PCh-BSA. Purified C1q (Calbiochem, 10 µg/ml was coated onto 96 well microtiter plates (Immulon 4 HBX, Thermo Fisher Scientific) at 4°C overnight. Plates were incubated with BSA (3% w/v) and Tween 20 (0.05% v/v) in PBS for non-specific site blocking at room temperature for 2 hours. Serum was obtained from healthy donors under informed consent at London School of Hygiene and Tropical Medicine (ethical approval 10672/RR/3680). Serum was diluted (1:20 v/v) with CRP (1 µg/ml) and BSA (1% w/v) in veronal-buffered saline (VBS) with 0.15 mM CaCl_2_ and 0.5 mM MgCl_2,_ with or without EDTA (10 mM). CRP ligand (fPPG, PCBSA) was added at a range of concentrations (0-3 µg/ml), and the plate was incubated at room temperature for 1 hour. Detection step was performed with a sheep anti-CRP conjugated with horseradish peroxidase (1:2000).

#### 2.7.2 3d deposition assay

To assess C3 convertase formation, an existing protocol to detect the C3 convertase downstream product C3d was adapted (18). *L. infantum* or *L. mexicana* fPPG (4 µg/ml) or PCh-BSA (0.4 µg/ml) was diluted in PBS and immobilised on 96 well microtiter plates (Immulon™ 2 HB 96-Well Microtiter EIA Plate, ImmunoChemistry Technologies, LLC). Ligand concentration was chosen to allow equivalent CRP binding as determined by previous CRP-ligand ELISA.

All additions were done with the reagents, serum and microtiter plate on ice. Donor serum was added to the wells (1:100 dilution) in GVBSCaMg (VBS with 0.2% v/v gelatin, 0.15 mM CaCl_2_ and 0.5 mM MgCl_2_), or GVBSEDTA (VBS with 10mM EDTA) or GVBSEGTAMg (VBS with 10mM EGTA plus 0.5 mM MgCl_2_) with or without additional purified CRP (0.4 µg/ml). Plates were brought up to a uniform temperature in a water bath (37°C) and incubated for 20 minutes. For C3d detection, plates were incubated with primary biotinylated anti-C3d antibody (1:1000) followed by HRP conjugated streptavidin (1:15000) in HBSC-BSA.

#### 2.7.3 iC3b deposition assay

Assay was performed as for C3d assay but plates were washed and iC3b detected with 1 µg/ml biotinylated antibody to neoepitope of iC3b (Quidel) followed by streptavidin-HRP (Invitrogen).

### 2.8 Immunofluorescent (confocal) microscopy

10^6^ *lpg1-/-* and WT parasites were washed with PBS and incubated in M199 medium containing 10 µg/ml CRP, control media only or media with CRP and 15 mM EDTA for 1 hour at room temperature. Parasites were centrifuged and fixed in 4% (w/v) PFA, washed and 2.5 x 10^5^ parasites, air dried onto slides. Slides were rehydrated and stained with rabbit anti human CRP (Calbiochem) followed by anti-rabbit IgG FITC (Dako) both for 1 hour with washing between stages. The slides were dried and counterstained with vectorshield + DAPI and coverslips attached. Slides were visualised using the Zeiss LSM880 confocal microscope.

## 3 Results

### 3.1 *L. mexicana* PPG binds to CRP

**Figure 1:**
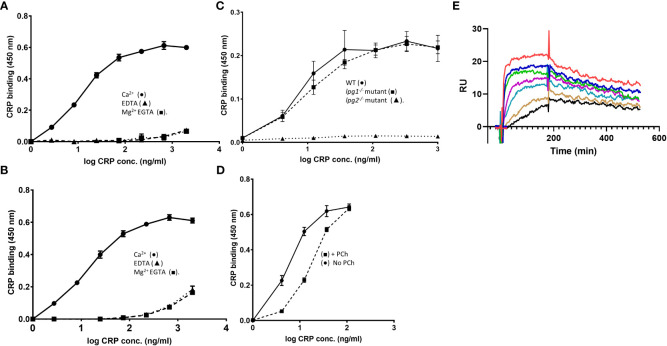
**(A–E)** CRP binds to *L. mexicana* fPPG. **(A)** CRP binds to fPPG in a calcium but not magnesium dependent manner. **(A)** *L. mexicana* wild-type fPPG (1 μg/ml) was immobilised on microtitre plates and biotin-conjugated CRP (0- 2.0 μg/ml) was added in the presence of Ca^2+^ (0.5 mM) (•); EDTA (10 mM) (▲) or Mg^2+^ (0.5 mM) EGTA (10 mM)(■). CRP binding was detected using HRP conjugated Streptavidin (1:15000) and TMB substrate (OD 450 nm). **(B)** Serum components do not compete with CRP binding. As in **(A)** but 5% (v/v) CRP depleted serum was added. **(C)** CRP binding to fPPG is independent of LPG. Biotinylated CRP (0 - 1.0 μg/ml) binds to immobilised *L. mexicana* fPPG coated at 0.3 μg/ml from WT (•) and *lpg1^-/-^* mutant (■) but not *Ipg2^-/-^* mutant (▲). **(D)** PCh competition inhibits CRP binding to fPPG. As in **(A)** but in the presence (■) or absence (•) of added PCh (10 mM). n=3. Error bars represent standard deviation. **(E)** Biosensor analysis of CRP binding to *L. mexicana* fPPG biotin immobilised onto neutravidin surface. 185 RU of neutravidin captured 340 RU of biotinylated fPPG. CRP (0.6- 40 μg/ml) was added for 3 minutes followed by 5 minutes dissociation. Traces show dose response of binding at two-fold dilutions after control flow cell subtraction.

Purified fPPG was heterogeneous and high molecular weight as previously described (23). This material was adsorbed onto a microtitre plate and CRP showed strong binding with a half maximal binding seen at 30 ng/ml (**Figure 1A**) with significant binding detected at less than 1 ng/ml CRP. This binding was not inhibited by the presence of 5% (v/v) CRP-depleted serum indicating a lack of competition by other serum proteins at any CRP concentration (**Figure 1B**). Although most LPG is removed at the hydrophobic interactions stage of purification, to confirm that CRP binding was not due to contamination by small quantities of LPG we also showed binding to PPG from a mutant lacking LPG (*lpg1^-/-^*) (**Figure 1C**). In contrast, the same preparations from the LPG2 mutant that lacks all addition of phosphoglycan to any protein or lipid showed no binding of CRP (**Figure 1C**). As expected for an interaction with the major ligand binding site on CRP, the relatively lower affinity ligand soluble monomer phosphorylcholine (PCh) significantly inhibits CRP-binding in a competitive manner (**Figure 1D**). The binding was inhibitable completely with either EDTA or EGTA Mg (**Figures 1A, B** **)** consistent with the calcium dependent binding site seen previous for binding of CRP to LPG (11, 14).

### 3.2 Kinetics of CRP PSG interaction by SPR

Binding of CRP to immobilised *L. mexicana* fPPG (**Figure 1E**) was examined over a range of CRP concentrations utilising the calcium dependency to allow complete elution of CRP between associations. As previously reported for other ligands, CRP shows complex interaction kinetics as expected for a pentamer binding to a ligand that is also potentially multimeric (18). The analysis used a low density of fPPG on the surface to reduce interaction of surface bound molecules. This low ligand density allowed analysis using simple 1:1 binding model with good fit (**Figure S1A**).The off-rate (k_d_) was 1.57e^-3^ +/- 3x10^-5^s^-1^ and on rate 4.3 x10^5^ +/- 5 x 10^3^ M^-1^ s^-1^ Overall avidity was estimated at 3.6 x 10^-9^ M consistent with the nanomolar CRP concentrations at which CRP binds in plate assays. fPPG from *L. mexicana* LPG deficient (*lpg1^-/-^*) mutant with PPG but no LPG also bound in SPR experiments (data not shown).

### 3.3 The major CRP binding component of the purified fPPG is the ScAP component

CRP was also shown to bind to the fPPG when separated by electrophoresis and transferred to membrane and the membrane was probed with CRP. Binding in the resolving gel was consistent with the molecular weight of ScAP. Binding to the material in the stacking gel could be to the filamentous proteophosphoglycan or to the acid phosphatase as this is strongly associated with the fPPG (10).

**Figure 2:**
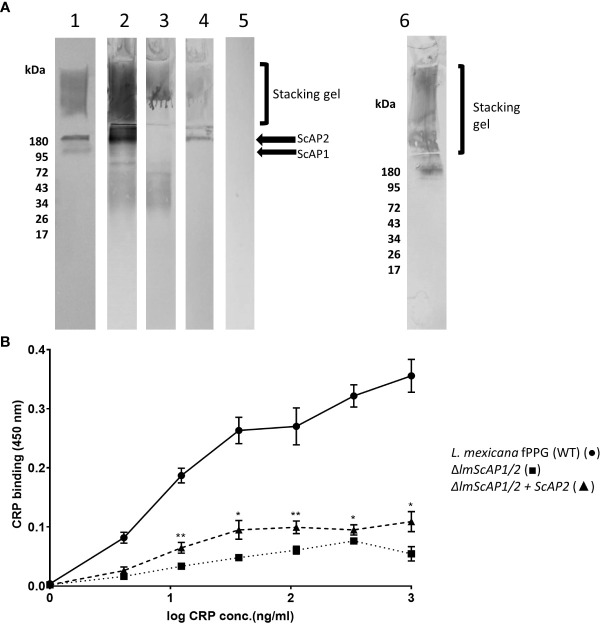
CRP binds to ScAP and fPPG from *L infantum* and *L. mexicana* **(A)** Binding of CRP to *L. mexicana* Wt, ScAP/-; and *ScAP/-ScAP2* fPPG separated by SDS gels and transferred to membrane. fPPG (3μg) was separated on 10% gels with stacking gel and transferred to PVDF membranes. Lane 1 membranes were probed with CA7AE and anti-mouse IgM to detect repeating disaccharide. Lanes 2 - 6 were probed with CRP and anti-CRP except Lane 5 which was a no CRP control. *L. mexicana* WT (Lanes 1, 2 and 5), △*lmScAP1/2* (Lane 3), △*lmScAP1/2* + *ScAP2* (Lane 4), *L infantum* WT probed with CRP (Lane 6). **(B)** ScAP provides the majority of CRP binding capacity in purified phosphoglycan. Dose-Response of binding of purified CRP (0 - 1.0 μg/ml) to immobilised *L. mexicana* fPPG (WT)(•), △*lmScAP*1/2 (■) △*lmScAP*1/2 + ScAP2 (▲). In both **(A, B)**, CRP binding was detected using rabbit anti-CRP antibody, HRP-conjugated goat anti rabbit antibody and TMB substrate (OD 450nm). n=4. Error bars represent standard deviation. Statistical comparison was performed for ScAP deficient versus add-back (△*lmScAP*1/2 vs △*lmScAP*1/2 + ScAP2). *p<0.01 ** p<0.001.

Therefore we used a mutant of *L. mexicana* that lacks both secreted acid phosphatases (Δ*lmScAP*1/2) and an add back of ScAP2 (22). The filamentous phosphoglycan is also a ligand as the mutant still retained binding in the stacking gel by blotting which could only be the fPPG (**Figure 2A**). Quantitation of relative binding by microtitre plate binding assay confirmed the reduced CRP binding to the ScAP deficient PPG (**Figure 2B**). Binding of CRP was partially restored in the add back as seen in both assays (**Figures 2A, B** **)**. In the add back, the amount of restored CRP binding was low, consistent with the plate binding assays but clearly associated with the ScAP2 that generated a band in the region of 200 kDa (**Figure 2A**). This suggested that the ScAP was a major ligand for CRP binding in the fPPG.

### 3.4 CRP binding to PPG from different *Leishmania* species shows similar high avidity but different ligand density

**Figure 3:**
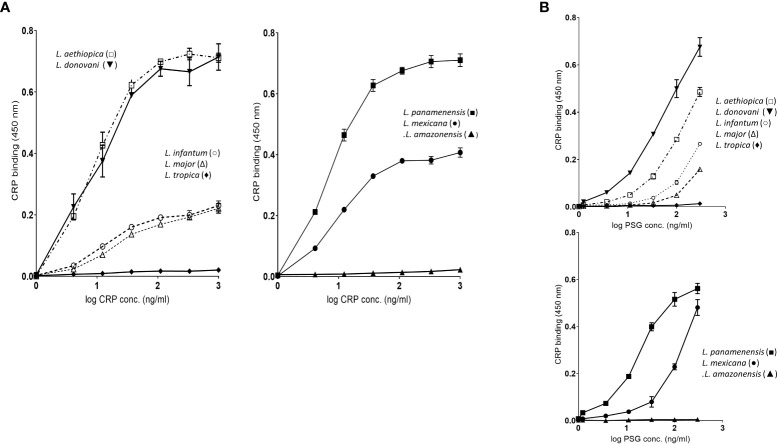
**(A)** fPPG from different *Leishmania* species exhibit widely varying CRP binding capacities. Dose response of binding of purified CRP (0 - 1.0 μg/ml) to fPPG (0.3 μg/ml) from different *Leishmania* species immobilised on microtitre plates. Bound CRP was detected using anti-CRP HRP. Left hand panels Old World species: Right hand panels New World Species. **(B)** CRP binding to varied fPPG coating concentrations with a constant CRP concentration (0.3 μg/ml). Highly substituted phosphoglycans (C3-galactose) *L tropica; L amazonensis*, Low or absent side chain substitution *L panamenensis, L donovani, L infantum, L mexicana*. *L aethiopica* has substitution on C-2 of mannose. Error bars represent standard deviation n=3.

We examined the binding to fPPG derived from different *Leishmania* species. The species included were 5 from the Old World and 3 from the New World and are shown in these groups in **Figures 3A** where binding was performed with a dose response of CRP to a constant PPG on the plate. In contrast, the panels in **Figure 3B** show the binding of a constant CRP to a range of ligand coating. The dose response of CRP binding to all parasites (**Figure 3A**) suggests that the ligand is of a similar high avidity for most parasites since the half maximal binding concentration (1-20 ng/ml) was similar for most species. High amounts of CRP binding represented a greater density of sites and binding to different species varied considerably at constant CRP (**Figure 3B**), for example, *L. donovani* had approximately 2 orders of magnitude more bound CRP than *L major*. There was no link between Old and New World species, nor did CRP binding appear different between species that cause different clinical presentation (visceral, cutaneous, or mucocutaneous). Previous studies by Thomas Ilg had characterised the proteophosphoglycan structures of different *Leishmania* species and correlated this with reactivity to monoclonal antibodies. A major factor in differences between species was side chain substitution, with strongest binding seen to those species that had little side chain substitution (discussed later).

The binding of CRP in plate assays was confirmed by the ligand blotting assays which showed a large amount of binding to material in the separating gel consistent with binding to ScAP (**Figure S2**). *L. donovani* and *L. panamenensis* PPG had particularly high binding capacity. *L. tropica* as in the plate assays displayed very little CRP binding activity.

### 3.5 CRP of other mammalian species also binds to repeating disaccharide

To determine if CRP interaction with phosphoglycan was restricted to human CRP or a feature of wider mammalian CRP we tested binding and showed that rat CRP bound strongly to the fPPG in a calcium dependent way (**Figure S3**). This analysis used a higher surface density of fPPG and a different immobilization chemistry on the chip surface. The rat CRP binding was calcium dependent but showed a lower number of available sites than human CRP but a slower off-rate. We saw little interaction with human SAP which was previously shown not to bind to LPG nor to hamster female protein an SAP homologue. A further control confirmed that these proteins purified on PCh were not binding to PCh as a monoclonal antibody to PCh (TEPC15) did not bind to fPPG (data not shown). Although CRP in mice is relatively low in concentration and sex dependent, the low concentrations required for binding are within physiological concentrations and we tested recombinant mouse CRP binding to PSG from different species. The order of binding was in broad agreement with that seen for human CRP (**Figure S4**).

### 3.6 CRP binding to PPG in lpg1^-/-^ parasites is located to the flagellar pocket

**Figure 4:**
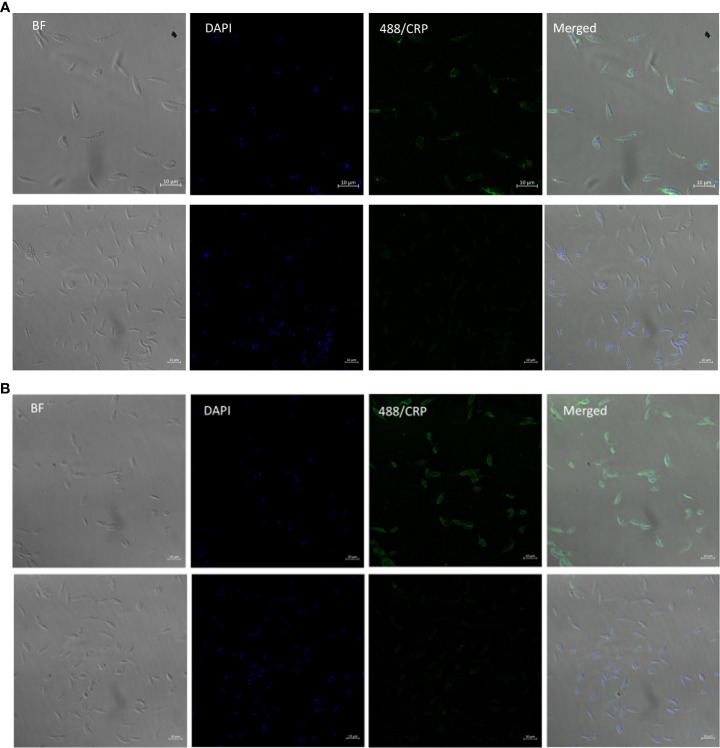
*L. mexicana* WT binds CRP all over surface in contrast to *lpg1*-/- mutant binding at flagellar pocket. **(A)** *L. Mexicana Ipg1*-/- and **(B)** *L. Mexicana* WT parasites were incubated with CRP and gently washed before fixing and CRP detected rabbit anti human CRP followed by anti-rabbit IgG FITC. DAPI was used as a counterstain. Images from left to right; phase contrast, DAPI, CRP immunofluorescence; merged Upper panels with CRP, lower panels without CRP.

In order to locate the CRP ligand in the LPG deficient and wild type parasites we performed immunofluorescent detection of ligand on live mutant and wild type parasites. The wild type showed strong and even binding across the surface of the parasite (**Figure 4B**) consistent with previous data for *L. donovani* (11). In contrast the *lpg1^-/-^* strain showed staining that was localised close to the flagellar pocket (**Figure 4A**). In order to observe this, the methodology was altered to reduce washing post CRP addition. If the parasites were centrifuged and resuspended in wash buffer then we observed numerous punctate staining particles separate from the parasites consistent with detached secreted PPG CRP complex (data not shown).

### 3.7 CRP binding to native sand fly derived proteophosphoglycan (PSG) is mainly to fPPG

**Figure 5:**
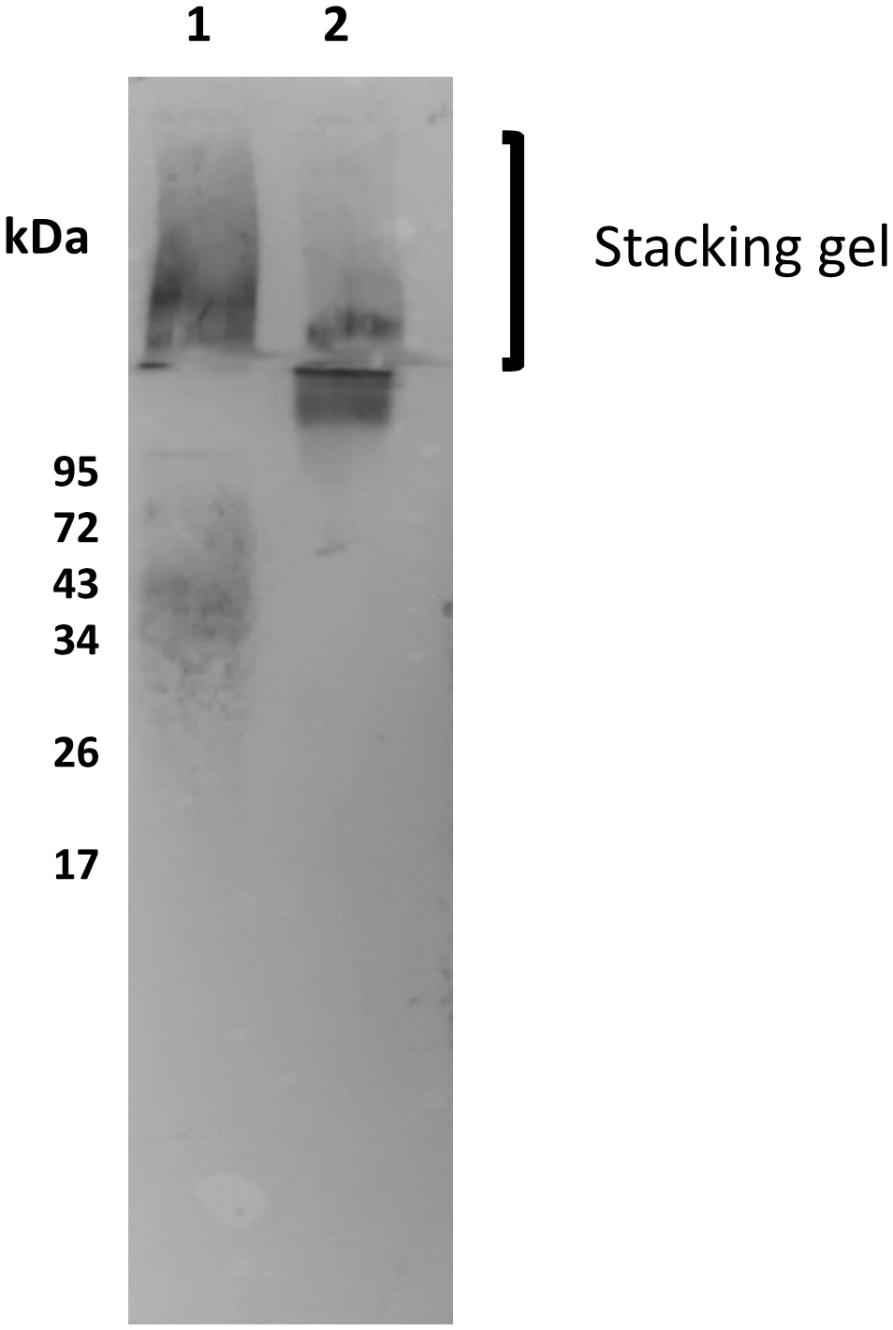
Native sandfly derived PSG and fPPG is a strong ligand for CRP. Sand fly PSG (0.5 μg/ml Lane 1) and purified fPPG (0.5 μg/ml Lane 2) from parasites were run on a 10% resolving gel with an extended stacking gel and transferred to PVDF membrane and probed with CRP-biotin (1 μg/ml) and streptavidin AP (1/500).

Although CRP binds to the purified cultured parasite fPPG, it was important to determine the binding to the physiological native promastigote secretory gel (PSG) found in infected sand flies. Previously it was shown that in *L. mexicana* the sand fly PSG was mainly composed of fPPG and there was little ScAP (26). PSG is associated with the parasites in the sand fly but also introduced at sand fly bites, therefore PSG was generated by microdissection and tested for binding to the CRP. Consistent with the limited ScAP presence we saw little binding to 200 kDa but very strong binding to the stacking gel and fPPG (**Figure 5**). This suggests that the main ligand in sand fly PSG was fPPG in contrast to the purified PSG from cultured parasites. Since the PSG from infected sand flies also contains *Leishmania* parasites the presence of LPG is detected as a broad band at around 50 kDa.

### 3.8 CRP binding to PSG activates complement

It was previously shown that *L. infantum* fPPG when injected with parasites caused an increased local survival of parasites (9) and *L. infantum* phosphoglycan is implicated in parasite virulence (5). fPPG and ScAP from *L. infantum* and *L. me*xicana bound CRP in a similar way (**Figures 2**, **3**). A proteophosphoglycan from *L. mexicana* amastigotes with similar carbohydrate structure to fPPG including repeating disaccharide was previously shown to deplete complement (20). Therefore, we chose fPPG from both these parasites to explore the ability of bound CRP to activate complement.

**Figure 6:**
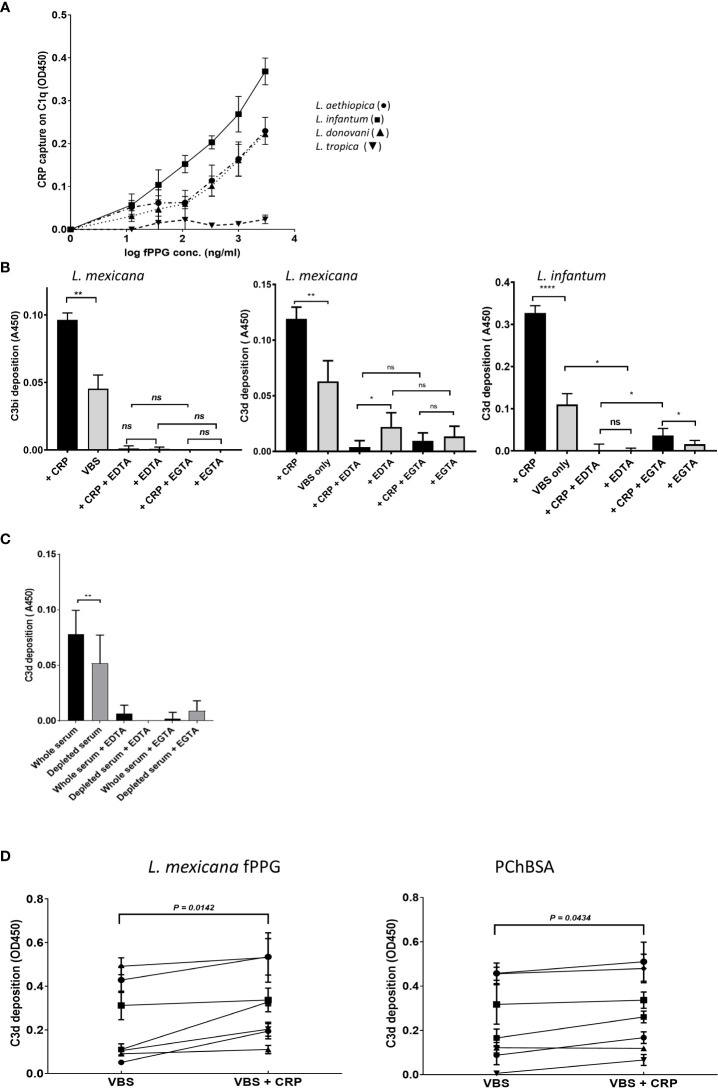
CRP contributes to fPPG mediated complement activation. **(A)** CRP that has bound to fPPG in fluid phase can be captured on C1q coated plates. Plates were coated with C1q. CRP (1 μg/ml) was added with different amounts of fPPG (0-3 μg/ml) and complex binding to the surface was detected with anti-CRP. Error bars represent standard deviation (n= 3) *L. aethiopica* (•) *L. infantum* (■) *L. donovani* (▲) *L. tropica* (▼). **(B)** fPPG from *L. mexicana* or *L. infantum* activates complement through the classical pathway and CRP. Microtitre plate assays of complement activation by fPPG from *L. mexicana* or *L infantum* coated at (2 μg/ml). Wells with immobilised fPPG were incubated with serum (1:100 dilution) for 20 mins at 37°C in the presence (filled bars) or absence (shaded bars) of additional purified CRP (0.4 μg/ml) in VBS Mg Ca buffer, or EDTA (5mM) or and Mg EGTA. Deposited C3bi or C3d was detected with biotinylated antibody followed by streptavidin HRP. C3bi mean and s.d, n=6. C3d Mean and s.d, n=4 *p<0.05; ** p<0.01; **** p<0.001; ns not significant..**(C)** Depletion of CRP in serum decreases the classical complement activation in response to *L. mexicana* fPPG. Methods as in **(B)**, but with whole serum and partially CRP depleted serum compared at the same dilution. **(D)** Variation in CRP mediated fPPG classical complement pathway activation in multiple donors. Added CRP (0.4 μg/ml) increases complement activation measured through C3d deposition as in **(A)** in response to fPPG coated at 0.4 μg/ml and PChBSA (0.1 μg/ml) and PChBSA in the presence and absence of additional purified CRP. Paired t test on 7 donors.

Using a capture assay with C1q attached to the plate and addition of serum containing CRP there was no observed binding of CRP to the C1q until fPPG addition which then generated a complex with the C1q. This showed a dose response as expected (**Figure 6A**). *L. tropica* did not lead to CRP binding to C1q consistent with the fPPG from this species failing to interact with CRP previously (**Figures 3A, B**). This interaction would be expected to lead to complement activation. This was confirmed when *L. mexicana* fPPG was used as a ligand in activation product assays that showed deposition of C3bi or C3d was by the classical pathway (**Figure 6B**). Classical pathway activation was increased by addition of CRP and reduced when CRP was partially depleted (**Figure 6C**). The depletion of CRP we achieved was only partial (50-95%) and the strong binding of CRP to its ligand suggests that the CRP would still have some contribution to the overall complement activation in depleted sera. Nevertheless, it was possible to demonstrate that a significant proportion of the activation of complement in serum was mediated by CRP. The lack of phosphoglycan and CRP ligand in *L. mexicana lpg2^-/-^* mutant resulted in a lack of CRP dependent complement activation (**Figure S5**). When CRP was added to individual sera and C3d activation was assayed there was a clear and consistent increase in activation apparent across different individual sera (**Figure 6D**). Activation was similar or greater than that for a PCh-BSA positive control, a known strong ligand for CRP mediated complement activation. The background activation by fPPG was however also variable suggesting that potentially a cross-reacting antibody was present in some sera.

## 4 Discussion

### 4.1 CRP binding to ScAP and proteophosphoglycan

Previously we demonstrated CRP binding to the extended repeating disaccharide of LPG. Here we have demonstrated the CRP can also bind with high avidity to multiple short disaccharides expressed on a backbone of a high molecular weight glycoprotein. Although previously we observed weak binding of CRP to soluble short repeats of the disaccharide in solution (14), the binding to multiple such structures on fPPG or ScAP was able to generate affinities similar to those seen previously to LPG. Although serum did not inhibit binding to the repeating disaccharide suggesting that CRP is the only major serum protein interacting with this epitope, CRP is not the only serum protein that binds to fPPG and the glycan structures present which include sidechain and cap structures. These include antibody and mannose-binding lectin (MBL) which have been detected by us using methods such as immunoprecipitation and others such as MBL which bound to amastigote PPG (aPPG) containing similar phosphoglycans to fPPG (20). The relative contribution of innate activators may vary considerably between *Leishmania* since we have shown that fPPG from *L. mexicana* and *L. infantum* bind much lower amounts of MBL than some other species such as *L. donovani*. The binding was consistent with the known binding properties of CRP in terms of calcium dependency and phosphorylcholine inhibition, in addition the kinetics showed that the avidity of binding was largely related to a slow off-rate (**Figure 1E**).

The observation that rat and mouse CRP binds *L. mexicana* fPPG is interesting, showing the phenomena is not restricted to human CRP although this observation needs extending to further species. Rat CRP has interesting binding features in that it binds to PCh but does not require the amino regions of the choline. In contrast, the HFP which is a homologue of SAP but also binds to phosphocholine in addition to phosphoethanolamine (27) did not bind.

For purified fPPG we observed that most of the binding was to the ScAP component despite being a minor component in terms of amount produced (22). fPPG having less binding for CRP is possible due to differences in the glycosylation of the PPG which has shorter Gal-Man repeats compared with ScAP and more frequent end capping that could mask the availability of the short repeat structure (28). However, the purification involved column separations and it is quite plausible that this process has enriched the lower molecular species at the expense of fPPG rich complexes trapped due to size by affinity media. The major ligand in PSG from sand flies was fPPG as very little binding was seen in resolving gels corresponding to ScAP. This does not rule out some binding in the stacker gel being due to ScAP but since fPPG is the major component this indicates that the major ligand in the gel is fPPG. The *fppg1* gene is present in most *Leishmania* and fPPG has been shown in *L. mexicana, L. major, L. amazonensis, Leishmania braziliensis, L. tropica* and *L. aethiopica* (29). The gel of PSG from infected sand flies also contains LPG as expected, revealed by CRP and CA7AE binding. CRP binds to the glycan of many PPGs but these may not always form filament networks as reported for *L. donovani* (30). Differences in size of fPPG and developmental changes have been shown for *L. major, L. mexicana* and *L. donovani* (31). There was no evidence for binding to other phosphoglycans such as aPPG or pPPG2 (32), since we did not examine amastigotes nor is aPPG a likely ligand since repeating disaccharide monoclonal LT6 does not bind (28).

### 4.2 Secretion of proteophosphoglycan

The secreted polymer fPPG has previously been shown to be located and polymer proposed to assemble in the flagellar pocket (30) so that we expected that fPPG would be localised here and this was demonstrated in LPG deficient parasites and shown to be quite different to the pattern for wild type LPG containing parasites where the LPG ligand is located over the whole parasite surface. Our data showed localisation of the secreted fPPG to the flagellar pocket in contrast to a previous study when *L. donovani* was analysed (33). In this study using an antibody generated to a protein part of fPPG the whole surface was also found to be labelled but there is little evidence elsewhere for the presence of fPPG bound to the surface. However, alternative carbohydrate substituted variants may be possible and there was heterogeneity in fPPG observed in our studies and elsewhere. Membrane PPGs (mPPG2) have been reported but do not appear to be a major feature in *L. mexicana* in terms of CRP binding as seen in the lack of CRP binding to the membrane in fluorescent micrographs of *lpg1^-/-^* parasites and consistent with structure discussed earlier.

### 4.3 Inverse correlation of binding and side chain substitution

Previous analysis of CRP binding to different *Leishmania* species showed strong binding to *L. donovani* LPG and others whilst some others only showed very weak or lack of binding to LPG. The binding was correlated inversely with the presence of LPG side chain substitution on the repeating disaccharide regions which hindered access to the disaccharide (34). Monoclonals generated against phosphoglycan structures show strong similarity in their ability to bind proteophosphoglycan and LPG from the same parasite (6, 35). In addition, comparison of the LPG and PPG side chains characterised by anion exchange HPLC show essentially the same pattern of structure for LPG and PPG but for *L. mexicana* the PPG contained much less side chain (23). Therefore, we hypothesized that CRP binding to PPG from different *Leishmania* would show the same pattern of binding as seen for LPG. *L. donovani* LPG is classified as type I with no side chain substitution and therefore we predicted that *L. donovani* PPG would be a strong binder as was found. Also in the *Viannia* subgenus, *L. braziliensis* and *L. panamenensis* have little side chain substitution (36) and *L. panamenensis* was found to be a good binder of CRP. In other reports *L. braziiensis* procyclic forms have side chain substitution but the metacyclic forms do not (37). The binding to *L. donovani* was also interesting because it had much longer repeat units (up to 32) on the ScAP analogous to that seen in LPG (38). *L. infantum* is devoid of side chain in the procyclic LPG form but has some in the metacyclic stage (39) and most strains of *L. infantum* are type I but variability is seen in certain strains (40).

*L. tropica* LPG (classified as type 2) has been shown to be highly substituted with almost all repeating units substituted at the C-3 position of the galactose with structures that are longer and often terminated with arabinopyranose (41). *L. amazonensis* has a high proportion of C-3 Gal positions substituted with chains of 2-3 sugars (42) and notably does not have significant binding to the CA7AE antibody that binds to repeating disaccharide repeats. Both these would suggest that CRP would have poor binding as indeed was demonstrated. Another LPG type 2 is *L. major* but this parasite shows considerable variation between strains for side chain substitution (41). It was observed that the side chain substitution of the *L. major* fPPG was less than that observed for the LPG from the same parasite, perhaps leading to significant CRP binding in these studies compared to *L. tropica* which did not bind.

*L. aethiopica* is known to be a type 3 LPG with substitution of a single mannose on the C-2 of mannose of the LPG repeats (41). It was previously suggested that this substitution site would have considerable effects on the conformation of LPG and its backbone which was supported by the failure of monoclonals to the repeating disaccharide to bind to this parasite LPG (43). CRP bound strongly to this fPPG, suggesting the different conformation of the repeating disaccharide allows CRP binding.

Phosphoglycans including the repeating disaccharide appear to be a feature across the *Leishmania* spectrum and most recently were confirmed in *Leishmania (Mundinia) enriettii* (44). However, there is no information about *Leishmania (Sauroleishmania) tarentolae*. The good correlation between low side chain substitution of *Leishmania* PPGs and strong CRP binding is consistent with the presence of the genes responsible for phosphoglycan synthesis, particularly those responsible for repeating disaccharide and side chain substitution (SCGs and SCGR families) in different species (45).

### 4.4 Complement activation by PSG

Given the role of PPG in depleting complement and that it aids parasite survival it was important to know if CRP activated complement at and beyond the C3 stage when binding to a phosphoglycan. Previously we showed that binding of CRP to another glycan structure terminating in a phosphorylcholine substituted onto an N glycan of filaria parasites effectively bound C1q and was able to generate C4b. However, this did not efficiently activate C2 and thus did not lead to an active C3 convertase but did functionally deplete complement (18). This property was related to the mobility of the glycan. For filaria, the lack of complement activation was important since any inflammatory response is damaging to survival, however infectious *Leishmania* is largely resistant to complement attack. It is not surprising therefore that the parasite generates material capable of strong activation of complement. PPG injected into a mouse could dramatically deplete complement by 90% within 30 minutes (20) and lasted for 24 hours. Whilst the mouse is unusual in that CRP is present at low levels even during inflammation the levels are sufficient to bind to the fPPG. Whilst there is little evidence for CRP or other innate proteins such as MBL or innate antibody having a role in killing metacyclic infectious promastigotes, these experimental systems may be too simplistic and do not rule out other roles for such activation in terms of altering survival *in vivo.*

It was previously shown that infection with *L. amazonensis* promastigotes caused a reduction in complement in mice. Complement-depleted mice showed a reduced inflammatory response and cell infiltrate and phagocytosis along with an increased parasite burden (46). A recent paper reported that *L. infantum* promastigotes activated both the classical and alternative pathways to different extents in different sera (dog, cat, and human) but each caused functional depletion of both pathways (47). Sandfly infection would be a better evaluation rather than footpad inoculation to evaluate fPPG and parasites and fPPG has been reported to be involved in several cell responses in the infected host (48), thus CRP may also have influence on responses other than complement or other responses indirectly through the complement effect. CRP makes a significant contribution to the activation of complement seen to the fPPG in human serum. Although significant data was generated with addition and depletion, our methodology for CRP depletion and addition was only partially effective with residual CRP in depleted sera and normal non supplemented sera still able to provide some activation. Thus, the relative contribution of CRP may be underestimated. Whilst normal CRP concentrations (less than 10 µg/ml) are sufficient to cause strong binding to PPG, concentrations are usually high in *Leishmania* patients (49).

These data demonstrate CRP-PPG interactions can have a role in parasite infection in sand flies when CRP is ingested as part of the blood meal. Effects of the blood meal are complex and have significant effects on infectivity and parasite differentiation and infectivity (49). This may be further complicated because insects have complement-inactivation mechanisms to protect their own epithelium which will affect the parasite susceptibility to blood meal derived complement (50). CRP may alter the physiological state of the phosphoglycan and/or the interaction with the parasite.

In conclusion, we found CRP is able to bind both ScAP and fPPG that make up PSG. CRP binding to PPGs from different *Leishmania* species is inversely correlated with side chain substitution, similar to previously seen for CRP binding to LPG. The CRP-PPG interaction is able to activate and deplete complement. Though the current study focuses on the role of CRP within the human host, these interactions may also be applicable to the sand fly vector.

## Data availability statement

The original contributions presented in the study are included in the article/**Supplementary Material**. Further inquiries can be directed to the corresponding author.

## Ethics statement

The studies involving human participants were reviewed and approved by the ethical committee of London School of Hygiene and Tropical Medicine. Written informed consent to participate in this study was provided by the participants.

## Author contributions

JR: Conceptualization, Funding acquisition, Investigation, Methodology, Resources, Supervision, Writing – original draft, Writing – review & editing. ES: Investigation, Writing – original draft. ED: Investigation, Writing – original draft, Writing – review & editing. JHS: Investigation, Writing – review & editing. MR: Conceptualization, Funding acquisition, Investigation, Project administration, Resources, Supervision, Writing – review & editing.
