# Supplementary material for: C-reactive protein binds to short phosphoglycan repeats of Leishmania secreted proteophosphoglycans and activates complement
Source: Front Immunol. 2023 Aug 31;14:1256205. doi: 10.3389/fimmu.2023.1256205 (PMC10500826; doi:10.3389/fimmu.2023.1256205)
Supplement: Supplementary file 1 [file DataSheet_1.pdf]

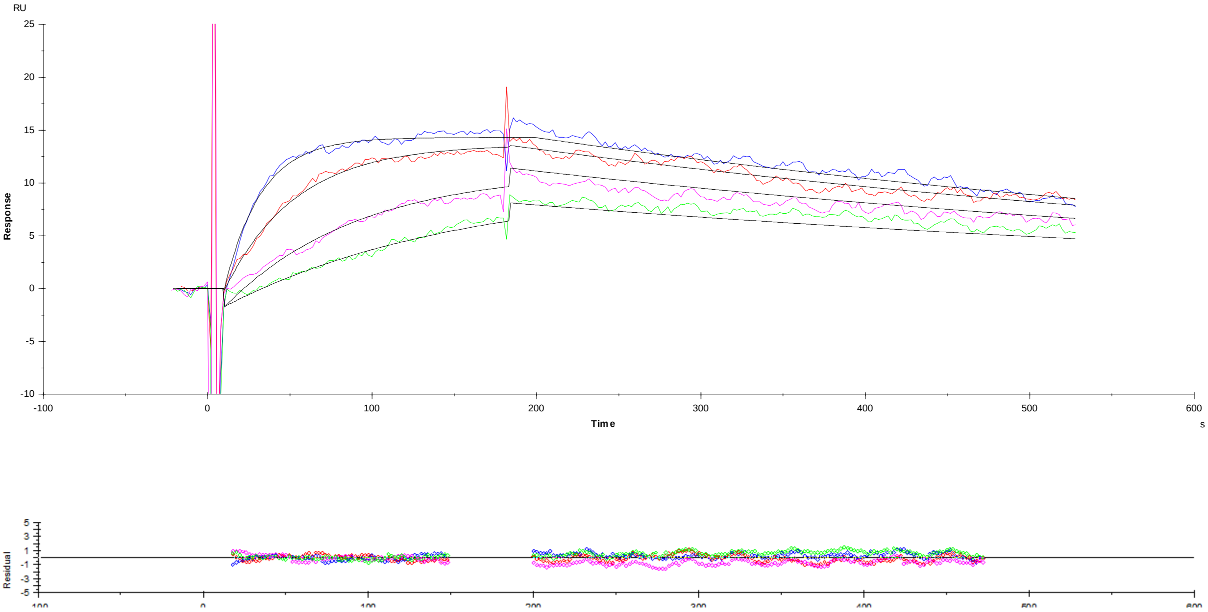

Figure S1. Biosensor analysis of CRP binding to *L. mexicana* PPG biotin immobilised onto neutravidin surface. 185 RU of neutravidin captured 340 RU of biotinylated PPG and CRP at 10-1.25 µg/mL was added for 3 minutes followed by 5 minutes dissociation. Upper panel shows actual SPR data and fit using 1:1 Langmuir model, and lower panel shows residuals of deviation from the fit. Kinetic analysis was performed using BiaEval global fit. Chi2 was 0.34 within the range of the noise and residuals small compared to the response. On-rate was 4.3 $x10^{5}$ +/- 5 x 10^{3} M^{-1} s^{-1} and off-rate $1.57e^{-3}$ +/- $3x10^{-5}s^{-1}$.

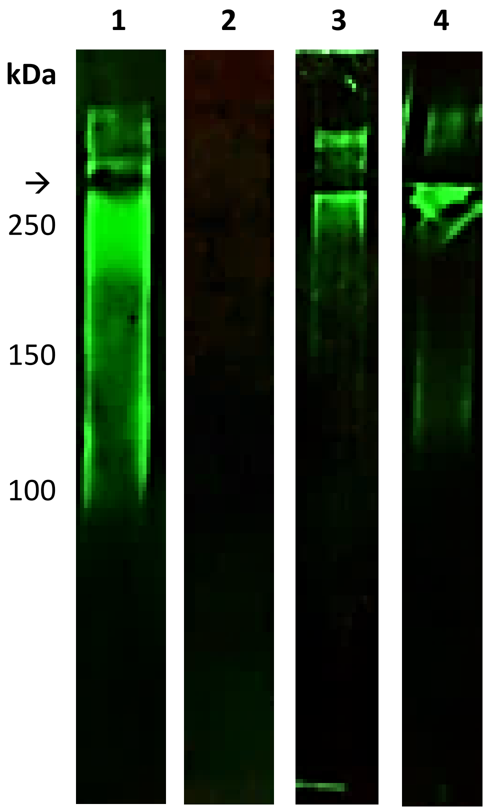

Figure S2. **fPPG from different *Leishmania* species exhibit widely varying CRP binding capacities.** CRP binding to 3 µg purified fPPG *L. panamensis* (Lane 1), *L. aethiopica* (Lane 3), *L. donovani* (Lane 4) but not *L. tropica* (Lane 2). fPPG was run on SDS with stacker and 10% resolving gel and transferred to PVDF and probed with CRP and rabbit anti CRP and IR800 anti-rabbit IgG. Images obtained from Storm are depicted in false colour.

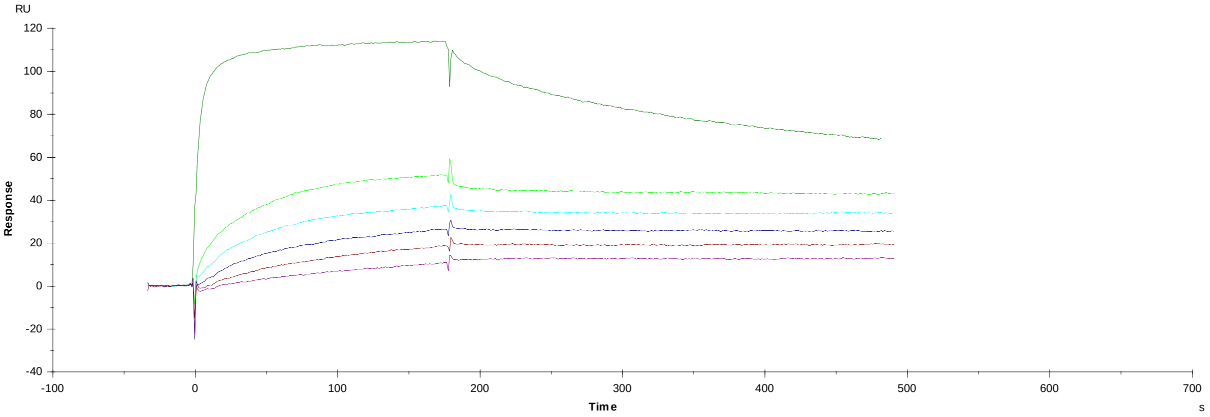

Figure S3. Rat CRP binding to *L. mexicana* fPPG

Surface was prepared using amine coupling of ultracentrigation prepared *L. mexicana* fPPG (1400RU) to a CM5 chip. Traces show from top purified human CRP (40 µg/ml) or purified rat CRP (40-2.5 µg/ml) flowed over the surface for 3 mins and dissociation followed for 5 mins before elution with EDTA. Following subtraction of control surface BiaEval was used to analyse kinetics. On-rates were approximately 10^5 $M^{-1}s^{-1}$ and off rates 5 $x10^{-5}$ s^{-1}.

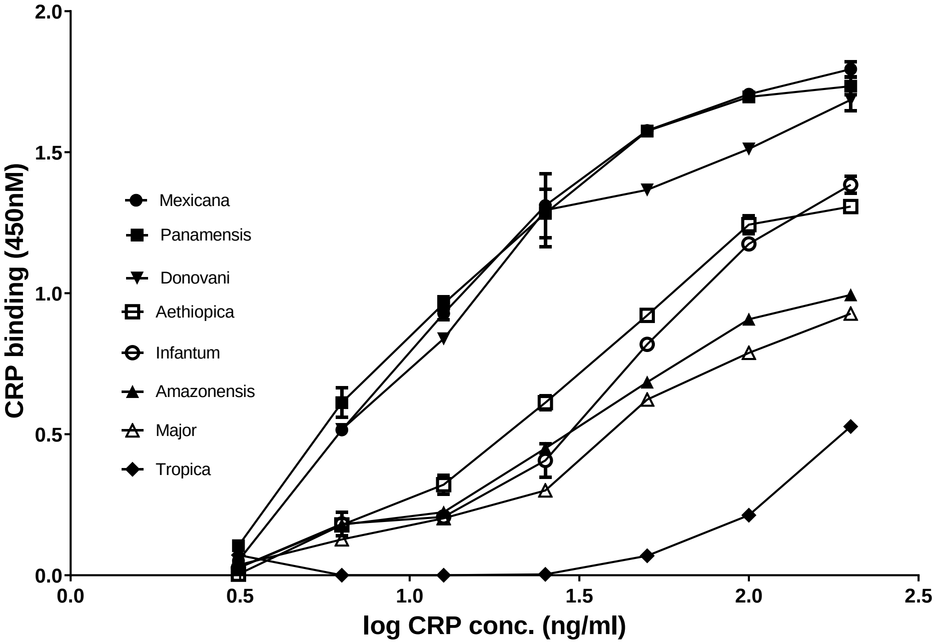

Figure S4. Mouse CRP binding to fPPG from different species. *L. tropica* (♦)*; L. major* (Δ)*; L. infantum* (○)*; L. donovani* (▼)*; L. aethiopica* (□)*; L. amazonensis* (▲)*; L. mexicana* (●)*; L. panamenensis* (■). Data was generated as in Figure 3 main paper. Error bars represent standard deviation n=3.

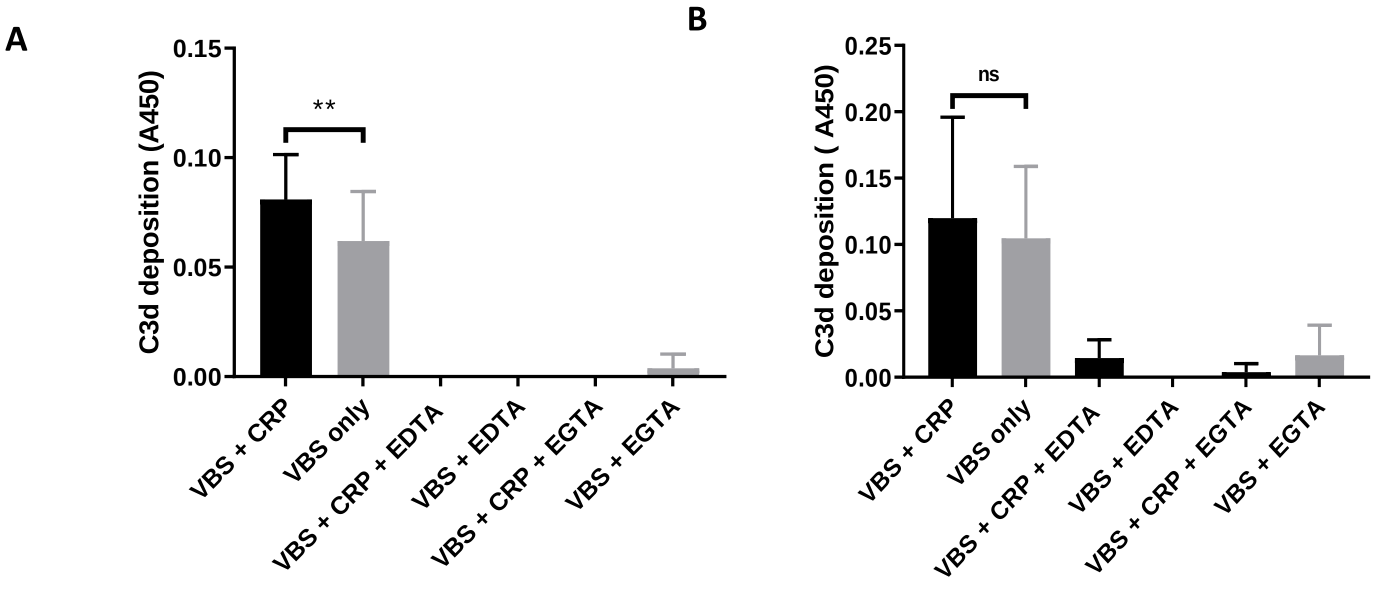

Figure S5. fPPG from *L. mexicana lpg1*$^{-/-}$ (A) but not *lpg2*$^{-/-}$ (B) shows CRP mediated complement activation. Data generated as in Figure 6B main text in plate assays using 2µg/ml fPPG coated wells. Mean +/- s.d. n =6. ** p<0.01. [illegible]
